# Do Sequential Cardiac Monitoring Methods Detect More Cases of Atrial Fibrillation in Post-Stroke Patients Compared to Non-Sequential Methods? A Retrospective Study

**DOI:** 10.7759/cureus.107327

**Published:** 2026-04-19

**Authors:** Mohammed Shariff, Talhah Chaudri, Shahid Kausar, Daniel Green, Ashim Banerjee

**Affiliations:** 1 Stroke Medicine, Russells Hall Hospital, Dudley, GBR; 2 Statistics, Aston University, Birmingham, GBR

**Keywords:** atrial fibrillation, cardiac monitoring, ecg, sequential, stroke

## Abstract

Background and purpose

There is currently a lack of formal guidelines on the optimal strategy for detecting atrial fibrillation (AF) post-stroke, with respect to the type and length of cardiac monitoring. The primary objective of this study was to determine if sequential methods of cardiac monitoring detect more cases of AF in post-stroke patients compared to non-sequential methods; the secondary objective was to determine if sequential methods of cardiac monitoring reduce the time taken to reach a diagnosis of AF in post-stroke patients compared to non-sequential methods.

Methods

A retrospective cohort study was conducted on data collected from 102 patients seen in the United Kingdom, at a district general hospital, at an outpatient stroke clinic, with a confirmed diagnosis of ischaemic stroke from 2019 to 2021. Data was collected on whether patients had sequential or non-sequential methods of cardiac monitoring and if AF was detected. Sequential cardiac monitoring was defined as one or more methods of cardiac monitoring (e.g., Holter monitoring, implantable loop recorder) with a stepwise progression to a longer form of cardiac monitoring if no AF was detected.

Results

A total of 41% of participants in the sequential monitoring group (number, n=18/44) had AF detected during the study period, compared to 22% in the non-sequential group (n=13/58) (hazard ratio 2.23, 95% confidence interval 1.02 to 4.85; p-value, p=0.04). 20% of the cases of AF in the sequential monitoring group (n=8.8/44) were detected 328 days earlier than 20% of AF cases in the non-sequential group (n=11.8/58) (p=0.047).

Conclusion

Sequential cardiac monitoring can detect more cases of AF and can reduce the time taken to reach a diagnosis of AF compared to conventional methods. The practicality and potential risks of implementing this method of sequential monitoring for all patients should, however, be considered.

## Introduction

Atrial fibrillation (AF) is the most common tachyarrhythmia, with a prevalence of 9% in those over the age of 75 [1]. The prevalence of AF in those who have had an ischaemic stroke has been found to be approximately 30% [2], although this figure is likely an underestimation due to the number of ‘silent’ cases that remain undetected. AF is also associated with poorer functional outcomes compared to other causes of stroke, such as intra- or extracranial large artery atherosclerosis [3].

Approximately 20% of patients presenting with ischaemic stroke already have a diagnosis of AF [4], and in those who do not, a simple bedside 12-lead electrocardiogram (ECG) will likely fail to detect those patients with paroxysmal AF, which is often subclinical [5]. These patients require more prolonged cardiac monitoring in order to detect these episodes of arrhythmia [5].

There is currently a lack of formal guidelines to advise clinicians on exactly how long monitoring should be carried out for to detect AF post-stroke and which method of prolonged monitoring should be used [6]. This lack of formal guidelines is reflected in clinical practice, where there is significant variability amongst senior clinicians on the type of inpatient and outpatient monitoring that is requested.

The primary objective of this study was to determine whether a stepwise approach (i.e., sequential cardiac monitoring) to detecting AF in post-stroke patients has a better detection rate compared to non-sequential methods. The secondary objective was to determine if sequential cardiac monitoring reduces the time taken to reach a diagnosis of AF in post-stroke patients, compared to non-sequential cardiac monitoring.

## Materials and methods

Study design

This was a retrospective cohort study that took place within the stroke department at a district general hospital. Data was collected between May and August 2023.

Participants 

Participant eligibility was determined using specific inclusion and exclusion criteria. Participants must have met all inclusion criteria to be included in the study, and were excluded if they met any one of the exclusion criteria.

The inclusion criteria included patients aged between 18 and 95 years; patients with a new diagnosis of ischaemic stroke (partial anterior circulation infarction [PACI], total anterior circulation infarction [TACI], or posterior circulation infarction [POCI]), confirmed either by a consultant stroke physician or on radiological imaging, including computed tomography (CT) imaging or magnetic resonance imaging; patients seen in a new outpatient clinic or in a follow-up clinic within the stroke department at the principal hospital by one of two different consultant stroke physicians; and patients who were seen in clinic between 1st January 2019 and 1st March 2021. 

The exclusion criteria included patients diagnosed with transient ischaemic attack (TIA), lacunar infarction, or primary haemorrhagic stroke; patients with symptomatic carotid artery stenosis (i.e., ipsilateral to the side of infarction) equal to or greater than 70% in those with PACI or TACI, confirmed on ultrasound carotid doppler imaging or CT angiogram of the carotid arteries; patients with a diagnosis of AF prior to the diagnosis of a new ischaemic stroke; and patients in whom AF was detected on a 12-lead ECG following the diagnosis of a new ischaemic stroke.

Variables

Eligible participants were divided into two groups based on whether they underwent sequential cardiac monitoring. The types of cardiac monitoring included in the study were 24-hour Holter monitoring, 5-, 7-, or 14-day Holter monitoring; 32-day Holter monitoring; and an implantable loop recorder (ILR).

Participants in the sequential cardiac monitoring group had at least one form of cardiac monitoring performed in a stepwise manner, progressing to a longer form of cardiac monitoring if no AF was detected. Participants in the non-sequential cardiac monitoring group had at least one form of cardiac monitoring performed, but there was no stepwise progression to a longer form of cardiac monitoring if no AF was detected. 

The outcome was defined as the detection of AF on a prolonged cardiac monitoring device, analysed and confirmed by a cardiac physiologist or cardiologist, or on a 12-lead ECG after follow-up with the stroke physician had concluded. 

For some patients, AF was detected after only a single type of prolonged cardiac monitoring (e.g., a five-day tape). If the intent of the clinician was to continue sequential cardiac monitoring in the event that AF was not detected, such patients were placed in the sequential cardiac monitoring exposure group. If the intent was to perform no further cardiac monitoring in the event of AF not being detected, then the patient was placed in the non-sequential cardiac monitoring exposure group. The intent was determined based on the clinician who was following up with the patient.

Potential confounders and effect modifiers

Potential confounders and/or effect modifiers in this study were identified as age, sex, type of stroke, past history of stroke or TIA, hypertension, hyperlipidaemia, diabetes, ischaemic heart disease (IHD), chronic kidney disease (CKD), left ventricular systolic dysfunction (LVSD; ejection fraction [EF] <45%), presence of patent foramen ovale (PFO), left atrial dilatation, smoking, obesity (body mass index, BMI >30 kg/m²), and alcohol excess. 

Data sources

A list of all patients seen in the outpatient clinic by two different consultant stroke physicians between 1st January 2019 and 1st March 2021 (inclusive) was compiled with help from the health informatics team at the hospital. The selection criteria were used to create a separate list of all eligible participants. Requests for cardiac monitoring for each participant were used to allocate participants to one of the two exposure groups. If a request for cardiac monitoring was not carried out or completed fully, the participant was allocated based on the monitoring that was requested rather than performed. 

Outcome data were then collected from the electronic patient record, including the types of cardiac monitoring performed for each participant, together with the date the results were analysed, the results, and whether AF had been detected or not. It was noted whether AF was detected during follow-up with the stroke physician or after follow-up had concluded. Data on potential confounders and/or effect modifiers were also collected from the electronic patient record, as well as clinic letters, discharge letters, admission documents, inpatient ward rounds, and echocardiogram reports.

Statistical methods

Data were analysed descriptively using frequency and percentage, or median with interquartile range (IQR) as appropriate. Parametric assumptions were assessed, such as assessing histograms or expected cell counts, and the most suitable hypothesis test was performed. 

Cox regression was used as the main analytical approach. The outcome of interest was whether AF was detected or not, and their time under observation started when they were admitted to the hospital or first seen in an outpatient clinic and ended in one of three scenarios: AF detected (event of interest), death (censored), or the study came to an end (censored), whichever came soonest. Kaplan-Meier curves were created to illustrate the event-detected rate with separate lines for the sequential monitoring status.

Variables of interest and potential confounders were entered individually to explore the univariable relationship with the outcome. Following this, a multivariable regression model was created. Multicollinearity was assessed via the variance inflation factor, and the proportional hazards assumption was assessed graphically.

For the primary analysis, in instances where data had not been collected for certain variables (such as smoking status, obesity, and excess alcohol), these observations were recorded as “no”. A sensitivity analysis was performed, removing these three variables from the multivariable analysis to explore the impact on the results and conclusions. A two-sided p-value (p) of 0.05 was considered statistically significant. The data were analysed using the Stata software, Version 18 [7].

Ethical considerations

Ethical approval was sought via completion of the Integrated Research Application System (IRAS) form and the local trust research and development department. Research Ethics Committee approval was deemed not necessary for this study.

## Results

Baseline data

Following application of the selection criteria, 102 participants were eligible to be included in the study. All those who were eligible were included in the final analysis. Table 1 demonstrates the baseline characteristics of the sample. 

**Table 1:** Baseline characteristics of the sample Key: $ = Mann-Whitney U Test; * = Chi-square test; £ = Fisher’s exact test N/A = data was unavailable; n = number of participants Values for all variables are presented as n (%), except for ‘age’ where values are presented as median (interquartile range)

Variable	Sequential cardiac monitoring		p-value
	No (n=58)	Yes (n=44)	
Age (years)	70.5 (60.8 to 77)	65.5 (54.5 to 74)	0.06^$^
Male	43 (74)	28 (64)	0.253*
Type of stroke			0.709*
Total anterior circulation infarct	2 (3)	3 (7)	
Partial anterior circulation infarct	33 (57)	23 (52)	
Posterior circulation infarct	23 (40)	18 (41)	
Past history of stroke or transient ischaemic attack	20 (34)	13 (30)	0.598*
Hypertension	44 (76)	21 (48)	0.003*
Hyperlipidaemia	31 (53)	29 (66)	0.205*
Diabetes	13 (22)	8 (18)	0.601*
Ischaemic heart disease	8 (14)	7 (16)	0.765*
Chronic kidney disease	15 (26)	8 (18)	0.358*
Smoker			0.730*
Yes	22 (38)	14 (32)	
Not applicable (N/A)	22 (38)	20 (45)	
Obesity			0.021*
Yes	15 (26)	13 (30)	
N/A	12 (21)	1 (2)	
Alcohol excess			0.268*
Yes	9 (16)	6 (14)	
N/A	34 (59)	20 (45)	
Left ventricular systolic dysfunction (ejection fraction <45%)	4 (7)	4 (9)	0.723^£^
Presence of patent foramen ovale	3 (5)	3 (7)	0.726*
Left atrial dilatation	20 (35)	15 (34)	0.967*

For some of the variables, there was a significant amount of data that was unavailable for some patients, and this is marked as ‘not applicable’ (N/A). For LVSD, PFO, and left atrial dilatation, there was a small amount of data that was unavailable for some of the patients, but for these variables, this data was marked as ‘no’ rather than ‘N/A’. 

Cardiac monitoring

The number of each type of cardiac monitoring requested in the sequential and non-sequential cardiac monitoring groups is summarised in Table 2.

**Table 2:** Number of each type of cardiac monitoring that was requested in the non-sequential and sequential cardiac monitoring groups Values are presented as the number of participants, n (%)

Type of cardiac monitoring	Non-sequential cardiac monitoring	Sequential cardiac monitoring
24-hour Holter monitor	17 (29)	12 (27)
5-, 7-, or 14-day Holter monitor	56 (97)	41 (93)
32-day Holter monitor	3 (5)	31 (71)
Implantable loop recorder	0 (0)	19 (43)

All the 24-hour Holter monitors that were requested were attached and remained attached for the duration of the monitoring period in both exposure groups.

For 5-, 7-, or 14-day Holter monitoring, all monitoring in the non-sequential cohort that was requested was attached; however, one did not remain attached for the duration of the monitoring period (i.e., only two days were recorded for a five-day Holter). In the sequential monitoring cohort, one patient (2.4%) did not attend their appointment to have the monitoring attached, and a further three patients (7.3%) did not have the full duration of their monitoring (for one patient, the reason was an adverse skin reaction to the electrode pads used for the Holter monitor).

For 32-day Holter monitoring, in the non-sequential monitoring group, one out of three patients (33.3%) did not attend their appointment to attach the monitor, compared to two out of 31 patients in the sequential monitoring group (6.5%). Eight patients in the sequential monitoring (25.8%) group had a shorter duration of monitoring than the anticipated 32 days.

Out of 19 patients in the sequential monitoring group who had an ILR requested, four of them (21.1%) declined ILR insertion either at the time of requesting or at a later date. One patient out of 19 (5.3%) was referred for ILR insertion but had yet to have their ILR inserted at the time of data collection.

Detection of AF 

The number of cases of AF detected in the sequential cardiac monitoring group was 18 out of 44 (41%) compared to 13 out of 58 (22%) in the non-sequential cardiac monitoring group (p = 0.044 [chi-square test]). 

All patients in whom AF was detected in the sequential monitoring group had it detected during follow-up with their stroke physician. In the non-sequential monitoring group, 7 out of 13 patients (53.9%) who had AF detected had it detected after follow-up with their stroke physician had ended, on 12-lead ECGs that were performed during hospital admission for reasons other than their primary event of ischaemic stroke.

Multivariable Cox regression analysis

A multivariable Cox regression analysis was undertaken using the following variables: sequential cardiac monitoring (the predictor variable), age, sex, hypertension, diabetes, IHD, smoking status, obesity, alcohol excess, and LVSD. Not all potential confounders were adjusted due to limited sample size, and hence the variables of interest chosen for analysis were based on a literature search [8-10] and expert opinion from a consultant stroke physician prior to analysis of the results.

Table 3 demonstrates the hazard ratios and confidence intervals for the multivariable Cox regression analysis. 

**Table 3:** Multivariable Cox regression analysis

Variable	Hazard Ratio	95% Confidence Interval	p-value
Sequential cardiac monitoring	2.21	1.00 to 4.88	0.049
Age	1.04	1.00 to 1.08	0.038
Male sex	0.74	0.33 to 1.65	0.460
Hypertension	0.81	0.36 to 1.84	0.615
Diabetes	0.75	0.26 to 2.19	0.600
Ischaemic heart disease	0.71	0.21 to 2.41	0.581
Smoker	0.81	0.34 to 1.93	0.629
Obese	0.90	0.32 to 2.57	0.850
Alcohol excess	1.32	0.42 to 4.22	0.635
Left ventricular systolic dysfunction (ejection fraction <45%)	1.98	0.54 to 7.35	0.305

Compared to those who underwent non-sequential cardiac monitoring, those who had sequential cardiac monitoring had a significantly increased chance of AF detection of 121% at any point during follow-up (hazard ratio, HR; 2.21, 95% confidence interval, CI 1.00 to 4.88; p=0.049), when accounting for the other variables included in the analysis. There was also a significant 4% increase in the detection of AF with each increase in unit of age at any particular time during follow-up (HR 1.04, 95% CI 1.00 to 1.08; p=0.038).

Sensitivity analyses

Due to the high number of unavailable data for smoking, obesity, and alcohol excess variables, a sensitivity analysis was undertaken that excluded these variables from the multivariate analysis. These results are summarised below in Table 4.

**Table 4:** Sensitivity analysis with exclusion of ‘smoking’, ‘obesity’, and ‘alcohol excess’ from the multivariable Cox regression analysis

Variable	Hazard Ratio	95% Confidence Interval	p-value
Sequential cardiac monitoring	2.23	1.02 to 4.85	0.044
Age	1.04	1.01 to 1.08	0.017
Male sex	0.78	0.36 to 1.68	0.522
Hypertension	0.81	0.35 to 1.84	0.610
Diabetes	0.75	0.27 to 2.06	0.574
Ischaemic heart disease	0.67	0.21 to 2.14	0.499
Left ventricular systolic dysfunction (ejection fraction <45%)	1.97	0.56 to 6.92	0.292

At any point during the study period, sequential cardiac monitoring increased the detection of AF by 123% compared to non-sequential cardiac monitoring (HR 2.23, 95% CI 1.02 to 4.85; p=0.044). 

Kaplan-Meier curve 

**Figure 1:**
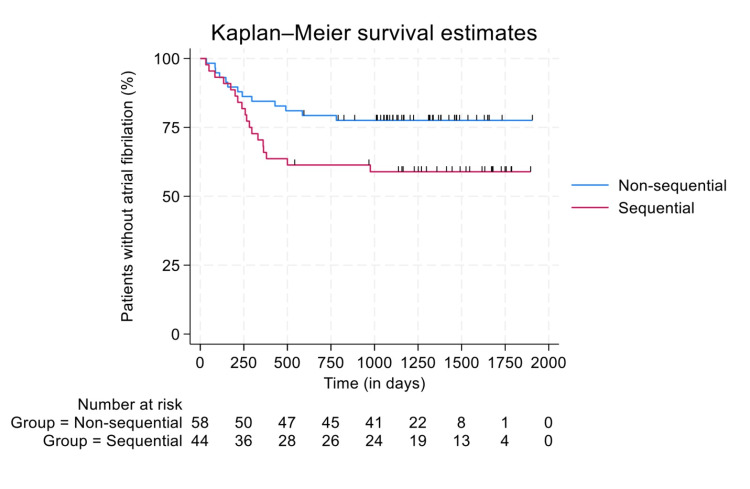
Kaplan-Meier curves demonstrating the percentage of patients without atrial fibrillation (X-axis) over time, in days (Y-axis) The blue curve represents the non-sequential cardiac monitoring group, and the red curve represents the sequential cardiac monitoring group.

Figure 1 demonstrates the percentage of patients without AF detected over time. The median follow-up time was 1157 days for the sequential cardiac monitoring group (IQR 286 to 1595) and 1113 days for the non-sequential cardiac monitoring group (IQR 786 to 1389). The proportion of patients without AF detected at 250 days was 81.8% in the sequential monitoring group (number of participants, n=36/44) and 86.2% in the non-sequential monitoring group (n=50/58). At 500 days, these proportions decreased to 63.6% in the sequential monitoring group (n=28/44) and 81.0% (n=47/58) in the non-sequential monitoring group. After 1000 days of follow-up, no further cases of AF were detected in either group.

The median survival time could not be calculated, as the percentage of patients without AF detected in either group did not reach 50%. The 20th percentile was 258 days for the sequential monitoring group, compared to 586 days in the non-sequential monitoring group.

Using the Log-Rank test, a p-value of 0.047 was generated for the Kaplan-Meier curves in Figure 1, indicating a statistically significant difference between the two curves over time.

## Discussion

Key results 

The primary objective of this study was to determine if sequential methods of cardiac monitoring detect more cases of AF compared to non-sequential methods in post-stroke patients. Analysis of the data demonstrated that sequential cardiac monitoring increases the number of cases of AF detected by 2.2 times compared to non-sequential cardiac monitoring in post-stroke patients. 

The secondary objective was to determine whether sequential cardiac monitoring reduces the time taken to reach a diagnosis of AF in post-stroke patients, compared to non-sequential cardiac monitoring. The Kaplan-Meier survival curve showed that 20% of the cases of AF were detected 328 days earlier in the sequential cardiac monitoring group compared to 20% of AF cases in the non-sequential group (p=0.047). 

It should be noted that for the final analysis, we included all cases of AF that were detected, whether it was during follow-up with a stroke physician or after follow-up had ended. Of the 13 patients in the non-sequential group in whom AF was detected, seven of them had AF detected after follow-up with their stroke physician had ended, on 12-lead ECGs that were performed during hospital admission for reasons other than their primary event of ischaemic stroke. If we had removed these seven cases of AF detected in the non-sequential group after follow-up had ended, it is likely that the evidence for sequential monitoring would have been more significant.

Interpretation

Our findings are supported by a large systematic review conducted by Sposato et al. [11]. A total of 50 studies were included, and cardiac monitoring methods across the studies were grouped into four phases, with each subsequent phase consisting of progressively longer cardiac monitoring methods. The proportion of patients who had AF detected increased by approximately 5% at each phase between phases 2 and 4, supporting the use of sequential cardiac monitoring methods to detect AF. 

A more recent study by Expósito et al. [12] had a similar study protocol to our own where 119 patients with cryptogenic stroke or TIA were included in a sequential cardiac monitoring programme consisting of a 24-hour ECG monitor, a 14-day external loop recorder, and an ILR. Using this sequential cardiac monitoring method, 36% of patients included had AF detected over a follow-up period of 36 months, a figure similar to our own finding of 41% of participants. The detection rate of AF with the use of a 24-hour ECG monitor was 4.2%, increasing to 18.75% with a 14-day external loop recorder, and then 21% with ILR insertion.

Although our study findings do suggest that sequential cardiac monitoring increases the detection rate of AF post-stroke and reduces the time taken to reach a diagnosis of AF, the potential harms associated with prolonged cardiac monitoring have to be considered, including poor compliance [5] and increased costs, as well as the practicality of implementing such an approach. The 2020 European Society of Cardiology guidelines for the diagnosis and management of AF [13] provide recommendations that for patients with acute ischaemic stroke or TIA without known AF, short-term ECG monitoring should be performed for 24 hours, followed by continuous ECG monitoring for at least 72 hours.

Beyond 72 hours, it is suggested that a selection of patients should undergo more intensive monitoring based on a number of proposed risk factors [5,13,14]. The recent 2023 National Clinical Guideline for Stroke for the United Kingdom and Ireland [15] advises that more prolonged sequential or continuous monitoring beyond 24 hours should be considered for those in whom a cardioembolic cause is suspected, and no other cause of the stroke has been found. Several clinical, biochemical, and electrocardiographic features have been postulated to be associated with increased risk of AF post-stroke [5,14], namely increased age, supraventricular extrasystole beats, atrial tachycardia, raised brain natriuretic peptide (BNP), increased left atrial diameter, and stroke aetiology. It would be sensible for clinicians to implement a sequential monitoring approach, but target more prolonged monitoring at those patients at increased risk of AF, and without any obvious cause of their stroke having been identified. This would increase the rate of AF detection in such patients whilst also reducing the need for more invasive monitoring methods [12].

A risk-stratification-based approach suggested by some experts [14] and guidelines [13] may be more practical and suited to working in a resource-limited healthcare system. Further research could be directed at directly comparing a sequential approach and a risk-stratification-based approach in terms of both AF detection and cost-effectiveness. Currently, there are no validated risk scores for predicting AF post-stroke, and those that have been proposed are of uncertain significance [16]. Development of a validated risk-assessment tool for AF post-stroke would be invaluable to stroke clinicians, and perhaps this should be the focus of future research to facilitate the development of robust guidelines in this field.

Limitations 

There are a few limitations to consider when interpreting the findings of this study. The retrospective nature of the study lends itself to being susceptible to missing data bias due to the historical nature of medical data records. This may have resulted in an overestimation of the effect of our predictor variable compared to if the full data for certain variables had been included in the final analysis. Furthermore, although we can establish a temporal relationship between the predictor and outcome variable with a cohort study, it is difficult to definitely prove a cause-and-effect relationship between the two using a cohort study alone, thus reducing the internal validity of the findings.

Secondly, confounding by indication may have occurred, as clinicians may have decided whether to perform sequential or non-sequential monitoring based on their own clinical experience and assessment of risk factors for that patient. This could imply that more cases of AF were detected in the sequential monitoring group simply because clinicians identified that such patients were at higher risk of AF, and therefore, they underwent sequential monitoring. 

With regard to participant selection, the sampling of participants from only one hospital and the resultant relatively small sample size mean that the sample of participants may not have been representative of the wider population of post-stroke patients to whom we wish to generalise the results, reducing the external validity of the study.

The measurements that were used to categorise participants into either the sequential monitoring or non-sequential monitoring exposure groups may have introduced misclassification bias into the study, which is a type of information bias [17]. Participants were allocated based on the type of monitoring that they received; however, if AF was detected after a single monitoring period, they were allocated based on the clinician that they were being seen by. This was an assumption that may have led to misallocation of participants, which could have been non-differential or differential depending on the percentage of misallocation in each group [17]. Non-differential misclassification reduces the true effect of the predictor variable, whereas differential misclassification can lead to either an increase or a decrease in the true effect [17]. This could therefore lead to invalid results; however, the assumptions were based on the real practising style of the clinicians, and as such, allocation would have been mostly accurate.

## Conclusions

This dissertation was carried out due to the lack of robust clinical guidelines for stroke physicians on the detection of AF post-stroke and the need for further research in this area. The aim was to investigate whether sequential cardiac monitoring improves the detection of AF in post-stroke patients. We found that sequential cardiac monitoring for post-stroke patients is associated with an increase in the number of cases of AF detected, as well as an association with a reduction in the time taken to reach a diagnosis of AF. These results are supported by recent studies that have also demonstrated that a sequential approach improves the detection of AF in post-stroke patients. However, it is important to consider our findings within the context of the study and recognise the limitations in applying our findings on a large scale. As a limited small-scale retrospective cohort study, the validity and generalisability of our findings require confirmation via further testing, either by prospective studies or, ideally, a randomised controlled trial. Nonetheless, our findings highlight the potential clinical value of adopting a more structured, sequential cardiac monitoring strategy in the post-stroke setting, particularly in improving early detection and facilitating timely management of AF. Future research should focus not only on confirming these results in larger, more diverse populations but also on evaluating the cost-effectiveness and practical implementation of such monitoring protocols within routine clinical practice.
